# Octocorals in the Gulf of Aqaba exhibit high photosymbiont fidelity

**DOI:** 10.3389/fmicb.2022.1005471

**Published:** 2022-11-25

**Authors:** Ronen Liberman, Yehuda Benayahu, Dorothée Huchon

**Affiliations:** ^1^School of Zoology, The George S. Wise Faculty of Life Sciences, Tel-Aviv University, Tel Aviv, Israel; ^2^The Interuniversity Institute for Marine Sciences, Eilat, Israel; ^3^The Steinhardt Museum of Natural History and National Research Center, Tel Aviv University, Tel Aviv, Israel

**Keywords:** Cp23S, ITS2, mesophotic coral ecosystems, Octocorallia, Symbiodiniaceae, SymPortal

## Abstract

Symbiotic associations, widespread in terrestrial and marine ecosystems, are of considerable ecological importance. Many tropical coral species are holobionts, formed by the obligate association between a cnidarian host and endosymbiotic dinoflagellates of the family Symbiodiniaceae. The latter are abundant on coral reefs from very shallow water down to the upper mesophotic zone (30–70 m). The research on scleractinians has revealed that the photosymbiont lineages present in the cnidarian host play an important role in the coral’s ability to thrive under different environmental conditions, such as light regime and temperature. However, little is known regarding octocoral photosymbionts, and in particular regarding those found deeper than 30 m. Here, we used ribosomal (ITS2) and chloroplast (23S) markers to uncover, for the first time, the dominant Symbiodiniaceae taxa present in 19 mesophotic octocoral species (30–70 m depth) from the Gulf of Aqaba/Eilat (northern Red Sea). In addition, using high-throughput sequencing of the ITS2 region we characterized both the dominant and the rare Symbiodiniaceae lineages found in several species across depth. The phylogenetic analyses of both markers were in agreement and revealed that most of the studied mesophotic octocorals host the genus *Cladocopium*. *Litophyton* spp. and *Klyxum utinomii* were exceptions, as they harbored *Symbiodinium* and *Durusdinium* photosymbionts, respectively. While the dominant algal lineage of each coral species did not vary across depth, the endosymbiont community structure significantly differed between host species, as well as between different depths for some host species. The findings from this study contribute to the growing global-catalogue of Cnidaria-Symbiodiniaceae associations. Unravelling the Symbiodiniaceae composition in octocoral holobionts across environmental gradients, depth in particular, may enable a better understanding of how specialized those associations are, and to what extent coral holobionts are able to modify their photosymbionts.

## Introduction

The mutualistic relationship between the cnidarian host and its endosymbiotic dinoflagellate algae of the family Symbiodiniaceae is vital for the persistence and continuity of coral reefs (Rowan and Knowlton, 1995; Berkelmans and Van Oppen, 2006; LaJeunesse et al., 2018). Through inorganic carbon fixation, the photosynthetic symbionts provide their host with sugars and amino acids, a crucial asset in oligotrophic seas such as the Red Sea (Muscatine and Porter, 1977). In return, the host provides the resident algae with light-rich conditions, a sheltered environment, and a supply of inorganic nutrients. Consequently, the cnidarian host and its associated dinoflagellate algae are critical partners in the diverse assemblage of eukaryotic and prokaryotic taxa that comprise the coral holobiont (Lesser et al., 2013; Aranda et al., 2016; Suggett et al., 2017).

The genetic diversity and composition of the algal symbionts has been studied using different molecular markers. Initially, Rowan and Powers (1992) applied restriction fragment length polymorphisms (RFLPs) of the nuclear small-subunit rRNA gene (SSU) to examine the phylogenetic relationships among the algal symbionts of different hosts. Subsequently, additional DNA regions were studied, including the less variable nuclear large-subunit rRNA (LSU: Lenaers et al., 1991; Rodriguez-Lanetty et al., 2001), the nuclear rRNA internal transcribed spacers (ITS1 and ITS2: LaJeunesse, 2001; Thornhill et al., 2007), the mitochondrial cytochromes *b* and cytochrome oxidase subunit 1 (*cytb* and *cox1*: Takabayashi et al., 2004; Sampayo et al., 2009), and the chloroplastic 23S rRNA and the gene encoding the D1 subunit of photosystem II (*cp23S* and *psbA*: Santos et al., 2002; LaJeunesse and Thornhill, 2011).

To date, 11 phylogenetic genera have been assigned to the family Symbiodiniaceae (LaJeunesse et al., 2018; Nitschke et al., 2020; Pochon and LaJeunesse, 2021). These were sampled from unrelated marine organisms such as foraminifers, ciliates, cnidarians, and mollusks, as well as from coral-reef sediment (Nitschke et al., 2022). Despite the lack of a standard molecular barcoding marker for Symbiodiniaceae identification, the ITS2 (~270 bp) is the most widely-used one to determine Symbiodiniaceae lineages at the genus level (e.g., Arif et al., 2014; Smith et al., 2020). This marker was adopted due to: (1) its large copy number (algal genomes enclose several 100 to several 1,000 copies of the rRNA cluster) (Saad et al., 2020)]; and (2) the existence of conserved flanking regions that enable the design of universal Symbiodiniaceae primers. It should also be noted that the fast rate of evolution of the ITS2 region appears to resolve the Symbiodiniaceae relationships at the genus level and even lower (Hume et al., 2018). However, the multi-copy nature of this marker creates a situation in which both intergenomic variants (i.e., polymorphism between algal individuals within a coral) and intragenomic variants (i.e., polymorphism between the ITS copies within an algal individual) may be simultaneously present within any coral sample. To identify the algal lineages present in a coral holobiont, it is critical to distinguish between these two sources of polymorphism. Specific analytical approaches have been developed to identify the confounding intragenomic diversity from the taxonomic diversity. In particular, profiling approaches, such as those implemented in SymPortal (Hume et al., 2019), enable diagnostic ITS2 type-profiles to be assigned, while examining the symbiotic algal composition at the highest possible resolution.

Coral-endosymbiotic algae interactions have been shown to be affected by heatwaves and other environmental stressors, which may result in the impairment and even breakdown of their mutualistic relationship (i.e., bleaching events; Hoegh-Guldberg and Bruno, 2010; Bahr et al., 2017; Hughes et al., 2018). In response to the increasingly recorded stressor events affecting coral reefs, research on the genetic and functional diversity of symbiodiniaceans has developed rapidly (LaJeunesse et al., 2010, 2018; Hume et al., 2019). Notably, it was found that the genetic lineages of the algal symbionts may modulate the phenotype of the holobiont in response to temperature rise or to poorly lit-environments, such as those affected by depth (Frade et al., 2008a; Cooper et al., 2011; Eckert et al., 2020; Smith et al., 2020). Consequently, an accurate determination of the Symbiodiniaceae diversity within corals is required in order to understand their hosts’ adaptive capabilities under changing environmental conditions.

Mesophotic coral ecosystems (MCEs) are coral-dominated communities found at a depth of 30–150 m. They act as an extension of the shallow-water reefs down to the lower edge of the photic zone (Kahng et al., 2010). MCE community structure is affected by the exponential decrease of light across depth (Kahng et al., 2019) and by the corals’ reliance on photoautotrophic carbon production *via* their endosymbionts (Rädecker et al., 2021). Most coral species at these depths are adapted to certain environmental conditions and can only thrive within a narrow depth range (i.e., “depth-specialists”; Liberman et al., 2022). A few corals, however, are able to thrive across a wider depth range (i.e., “depth-generalists”). The ability to host different symbiodiniaceaens across depth may benefit depth-generalists (Bongaerts et al., 2010; Ziegler et al., 2015). For example, the shallow-water population of the anthozoan *Stylophora pistillata* in the Gulf of Aqaba/Eilat hosts members of the genus *Symbiodinium*, whereas the mesophotic population is dominated by members of the genus *Cladocopium* (Scucchia et al., 2021). Similar shifts in Symbiodiniaceae diversity across depth exist in *Madracis pharensis* (Curacao: Frade et al., 2008a), *Seriatopora hystrix* (western Australia: Cooper et al., 2011), and *Montastraea cavernosa* (Belize: Eckert et al., 2020). However, changes in algal diversity across depth are not considered to be a general phenomenon, and some depth-generalist species exhibit similar Symbiodiniaceae composition across their entire depth of occurrence such as *Pachyderms speciosa* (Western Australia: Cooper et al., 2011), *Seriatopora hystrix*, and *Porites* spp. (Red Sea: Ziegler et al., 2015).

Despite the high abundance of zooxanthellate octocorals in MCEs (Bridge et al., 2012; Shoham and Benayahu, 2017; Benayahu et al., 2019), only a few studies have investigated the diversity of symbiodiniaceaens associated with octocorals (Goulet et al., 2019). Indeed, their diversity in MCE octocorals has not been the focus of any study to date. The genetic composition of Symbiodiniaceae inhabiting shallow-water octocorals has been examined in different biogeographical regions (Caribbean: Goulet and Coffroth, 2004; Hannes et al., 2009, Great Barrier Reef, Australia: LaJeunesse et al., 2004; Van Oppen et al., 2005; Ziegler et al., 2018, Red Sea: Barneah et al., 2004; Goulet et al., 2008). Most of the shallow-water octocoral Symbiodiniaceaen algae were studied using the RFLP or DNA fingerprint approaches (Goulet and Coffroth, 2003, LaJeunesse et al., 2004, Goulet et al., 2008, but see Van Oppen et al., 2005). These methods led to the suggestion that octocoral colonies are probably associated with members of a single genus. Recently, the algal composition of six shallow octocoral species was studied using high-throughput sequencing methods (Ziegler et al., 2018: *Bayerxenia* sp., *Isis* sp., *Lobophytum* sp. and *Xenia* sp.; Osman et al., 2020: *Sarcophyton trocheliophorum* and *Xenia umbellata*), which have also revealed members of a single Symbiodiniaceae genus associated with individual octocoral colonies.

Clearly, our current knowledge of the genetic structure of octocoral Symbiodiniaceae communities and of the more cryptic genetic identities is insufficient. Motivated by this lack of knowledge, the present study sought to (1) examine, for the first-time, the genetic lineages of Symbiodiniaceae of MCE octocorals from the Gulf of Aqaba/Eilat; and (2) compare the Symbiodiniaceae communities of depth-specialists across a depth gradient using both Sanger sequencing and high-throughput sequencing of the ITS2 with the SymPortal analysis framework. Our results revealed a pattern of conserved symbiont community along depth for most the examined species, indicating that host-symbiont specificity may drive Symbiodiniaceae diversity in the Gulf of Aqaba/Eilat octocorals.

## Materials and methods

### Collection of samples

In order to examine the genetic composition of Symbiodiniaceae in the Gulf of Aqaba/Eilat MCE, octocoral samples were collected from seven locations (Supplementary Figure S1) in two sampling campaigns during 2017–2018 (45–70 m) and in an additional one during 2019 (40–70 m) using a Remote Operating Vehicle (ROV: ECA H800) operated by Sam Rothberg R/V of the Interuniversity Institute for Marine Sciences in Eilat) and technical diving (Table 1). Shallow-reef selected taxa (0–21 m) were also collected from these locations during 2017–2020 using SCUBA diving (all samples were collected under permits issued by the Israel National Parks and Nature Reserves Authority: permits 2017/41761, 2017/41581, 2018/41893, 2018/42526, and 2018/41902). The octocorals host species were first assigned to species in the field based on their morphology. Later, the 70% ethanol preserved subsamples were taxonomically identified based on the relevant literature and on the octocoral reference collection at the Steinhardt Museum of Natural History, Israel National Center for Biodiversity Studies at Tel Aviv University. For subsequent Symbiodiniaceae sequence analysis, subsamples (1×1 cm) of the octocoral colonies were preserved in absolute alcohol and stored at −20°C.

**Table 1:** List of octocoral taxa used in the study, their collection depth, site, and total number of samples from shallow reefs and mesophotic coral ecosystems.

Species	Collection depth (m)	Collection site	Number of specimens
*Cladiella* sp.	Shallow: 3	Princess Beach	1*
*Klyxum unitomii*	Shallow: 3–6; MCE: 45–62	EAPC_C, Princess Beach	2 4
*Ovabunda* sp.	Shallow: 5–10; MCE: 40–50	IUI	5 5
*Xenia* sp.	MCE: 62	IUI	1*
*Briareum hamrum*	MCE: 55–60	IUI	2
*Anthelia glauca*	MCE: 55–58	IUI	2
*Sarcophyton glaucum*	MCE: 55–61	IUI, Princess Beach	4
*Sarcophyton auritum*	MCE: 38–40	EAPC_C, EAPC_N	2
*Lobophytum depressum*	MCE: 40–43, 56	IUI, Dekel Beach	3
*Rhytisma fulvum*	Shallow: 2–10; MCE: 38–45	IUI	7 7
*Litophyton* sp.	MCE: 38	IUI	1*
*Litophyton savignyi*	MCE: 38–45	IUI	6
*Litophyton arboreum*	Shallow: 3–11	IUI, Dolphin Reef	7
*Sinularia hirta*	MCE: 39, 58	IUI, Princess Beach	2
*Sinularia eilatensis*	Shallow: 10–17; MCE 58–66	IUI, EAPC_S, Dolphin Reef	6 6
*Sinularia vrijmoethi*	Shallow: 1–12; MCE: 40–50, 63	IUI, EAPC_S; IUI, Princess Beach	3 5
*Sinularia leptoclados*	Shallow: 3–5; MCE: 64–65	Princess Beach	2 2
*Sinularia mesophotica*	MCE: 60–67	EAPC_C, Dolphin Reef	3
*Sinularia loyai*	MCE: 38–40	IUI	2
*Sinularia querciformis*	MCE: 50, 67	Dekel Beach, Dolphin Reef	2
*Sinularia polydactyla*	MCE: 49	Dekel Beach	1*

IUI, interuniversity Institute for Marine Sciences in Eilat; EAPC, Eilat-Ashqelon Pipeline Company central beach, see Supplementary Figure S1 for exact locations. Asterisk indicates host species that were found only once in our surveys.

### Identification of algal symbionts

Genetic identification of the dominant Symbiodiniaceae at the genus and type levels was performed by sequencing two DNA markers: ITS2 and *cp23S*. DNA was extracted from preserved octocoral fragments using the PureLink DNA extraction kit (Thermofisher) according to the manufacturer’s instructions (*N* = 38, *N* = 31, for each marker, respectively). The genomic DNA region spanning the 5.8S and ITS2 of the Symbiodiniaceae sp. was amplified using the primers SYM_VAR_5.8S2 (5′-GAATTGCAGAACTCCGTGAACC-3′) and SYM_VAR_REV (5′-CGGGTTCWCTTGTYTGACTTCATGC-3′) (Hume et al., 2018). The chloroplast 23S gene was amplified using the primers 23S1 (5′-CGACGGCTGTAACTATAACGGTCC-3′) and 23S2 (5′-TTCACACAGGCCATCGTATTGAACCCAGC-3′) (Zhang, 2000). PCRs were performed in 25 μL total reaction volume containing 2 μl of DNA template (~10–100 ng), 2.5 μL of 10X ExTaqTM buffer, 2 μL of dNTPs supplied with ExTaq kit (2.5 mM each), 0.15 μL of TaKaRa ExTaqTM polymerase (5 units/μL), 5 μl of Betaine (5 M), 2.5 μl of each primer (5 pM), and 8.5 μL of sterile H_2_O. The PCR steps for the ITS2 region were performed according to Hume et al. (2018) and for the 23S region according to Santos et al. (2002). PCR products were purified using the ExoSAP (Thermofisher) approach, and directly sequenced on an ABI 3500xl genetic analyzer by the DNA Sequencing Unit of the George. S. Wise Faculty of Life Sciences, Tel Aviv University. In contrast to the chloroplast 23S, the ITS2 sequences usually revealed background noise, suggesting that more than one sequence may exist in each of these samples. Therefore, in order to determine whether depth-generalist species contain similar Symbiodiniaceae communities, the ITS2 intragenomic diversity of both shallow and MCE fragments was analyzed by high-throughput sequencing (Hume et al., 2018, see also below).

### High-throughput ITS2 sequencing

Genomic DNA from different octocoral host species (i.e., *L*. *arboreum* (*n* = 4), *L*. *savignyi* (*n* = 4), *Ovabunda* sp. (*n* = 8), *Rhytisma fulvum* (*n* = 12), *Sinularia eilatensis* (*n* = 8), *S*. *vrijmoethi* (*n* = 8), *S*. *leptoclados* (*n* = 5), and *S*. *mesophotica* (*n* = 3) were amplified with primers that included overhang adapters to fit the MiSeq platform (see Supplementary material). These PCR products were sent to Hy Laboratories Ltd., Israel for a second PCR using the Access Array tag for Illumina primers (Fluidigm Corporation, United States). This PCR added the index and adaptor sequences required for multiplexing and sequencing on an Illumina platform. The samples were pooled together and sequenced on an Illumina Miseq using a v2-500 cycle kit to generate 250 × 2, paired-end reads. The data were de-multiplexed by the Illumina software, and the de-multiplexed FASTQ files were then submitted to SymPortal for quality control and analysis (SymPortal.org; Hume et al., 2019; last accessed on November 2021).

### Data analysis

#### Analyses of sanger sequences

Phylogenetic trees for the ITS2 and *cp23S* sequences were reconstructed by combining newly obtained Sanger sequences and previously published Symbiodiniaceae sequences available in open-access databases. For the ITS2, we included all sequences available in the ITS2-DB database (Shi et al., 2021). This database provides a reference for consistent and precise genotyping of Symbiodiniaceae. For the *cp23S* marker we included all sequences from the database of Nitschke et al. (2020).

Sequences were aligned using MAFFT (Version 7) under the L-INS-I algorithm (Katoh et al., 2018). Unreliable alignment positions were identified using the GUIDANCE 2 webserver (Sela et al., 2015)^1^ under the same alignment parameter. Positions with a score below 0.93 were excluded. Phylogenetic relationships were reconstructed under the maximum likelihood (ML) criterion as implemented in the program IQ-TREE version 2.1.3 (Nguyen et al., 2015). The analyses were run with the options –m MFP –b 1,000 (i.e., ModelFinder + tree reconstruction+1,000 non-parametric bootstrap replicates). Model selection was performed using the Bayesian Information Criterion (BIC).

#### Symbiodiniaceae community composition analysis

Statistical analyses of Symbiodiniaceae diversity were conducted on SymPortal outputs in the R statistical environment (R Core Team, 2019; version 4.1.1), following the methods and code published by Eckert et al., 2020).^2^ To plot and examine ITS2 relative sequence abundance, the post-MED absolute abundance sequence count tables were used. To account for differences in sequencing depth among individual libraries, ITS2 type and type profiles reads were normalized using trimmed mean of M-values (TMM) in the package edgeR 3.14. This correction has been shown to effectively reduce false discovery rates and improve true positive rates (Pereira et al., 2018). To assess the statistical difference in ITS2 type assemblages between depths and between octocoral species, a PERMANOVA was performed with the “adonis” function from the package vegan_2.5–7 (Oksanen et al., 2020). For this purpose, we used the normalized ASV sequences which encompass copy number and intragenomic variability below the species level (Davies et al., 2022). The PERMANOVA analysis was run with 999 permutations using Bray-Curtis dissimilarities as distance metric. To determine whether depth and host species were significant factors, a nested-PERMANOVA was performed, with one factor as a fixed factor and the other as a blocking factor, and *vice-versa*, using the “adonis2” function in vegan_2.5–7. To compare the ITS2 type assemblages found in each host species, a pairwise PERMANOVA test was then performed, with the function “pairwiseAdonis” of vegan 2.5–7. A false discovery rate (FDR) correction was applied to the obtained *p*-values to account for the multiple comparisons. Finally, to identify which ITS2 type sequences had a significant effect on the differences found in the PERMANOVA tests, a similarity percentage test (SIMPER) was performed on the square-root of the normalized abundances, using the function ‘simper’ in vegan_2.5–7.

## Results

### Sanger sequencing of ITS2 and cp23S

**Figure 1:**
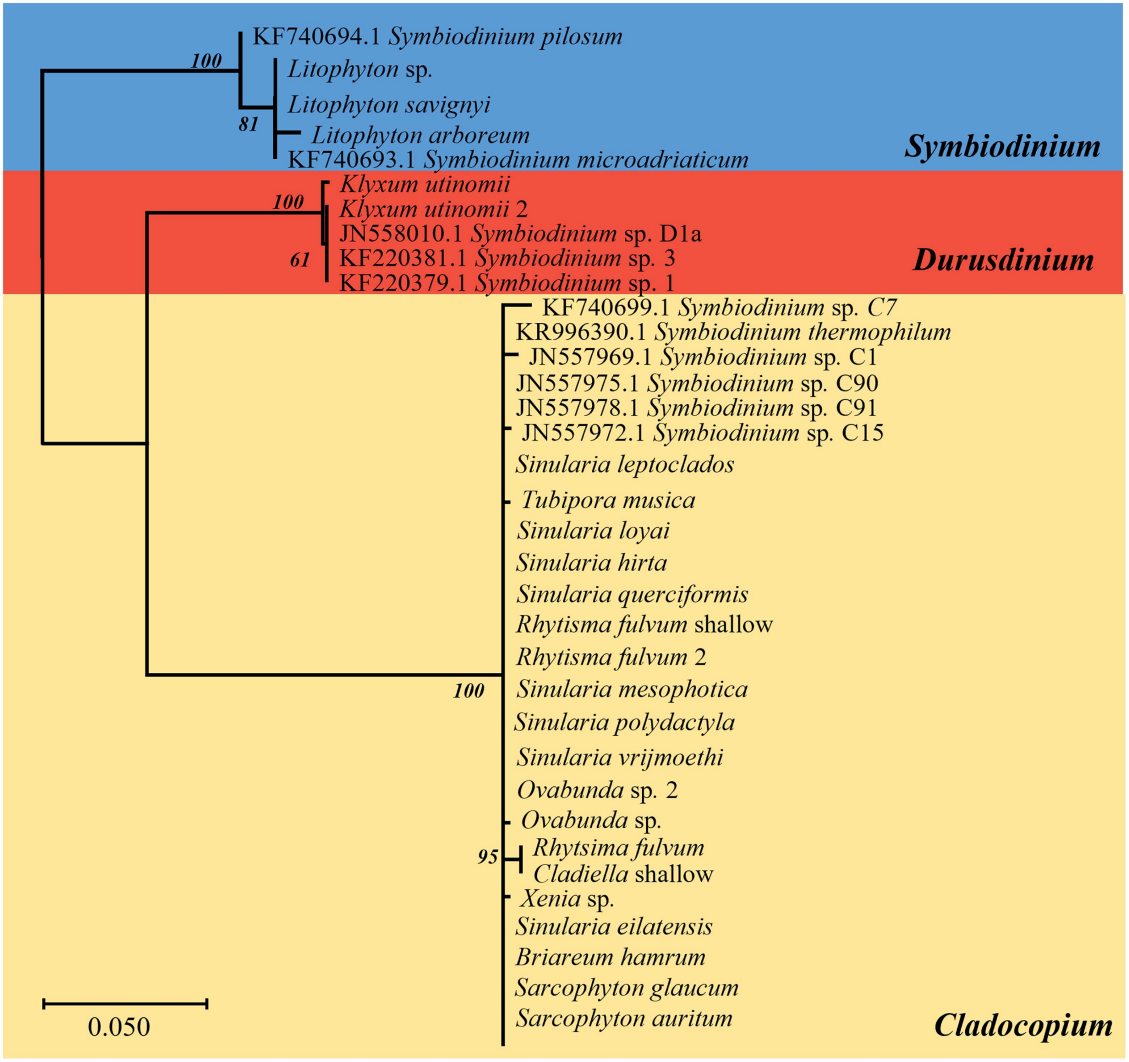
Phylogenetic relationship of Symbiodiniaceae based on chloroplast 23S rRNA sequences (500 bp) under the HKY + F model. The maximum likelihood tree was reconstructed with IQ-TREE 2.1.3. ML bootstrap support based on 1,000 replicates are indicated either above or below the corresponding branch. Colors represent different Symbiodiniaceae genera: blue *Symbiodinium* algae, red *Durusdinium* algae, and yellow *Cladocopium* algae. Species that presented a variation in the obtained sequence are indicated by the number 2. For the obtained new Symbiodiniaceae sequences, the host name is indicated rather than the algal type.

**Figure 2:**
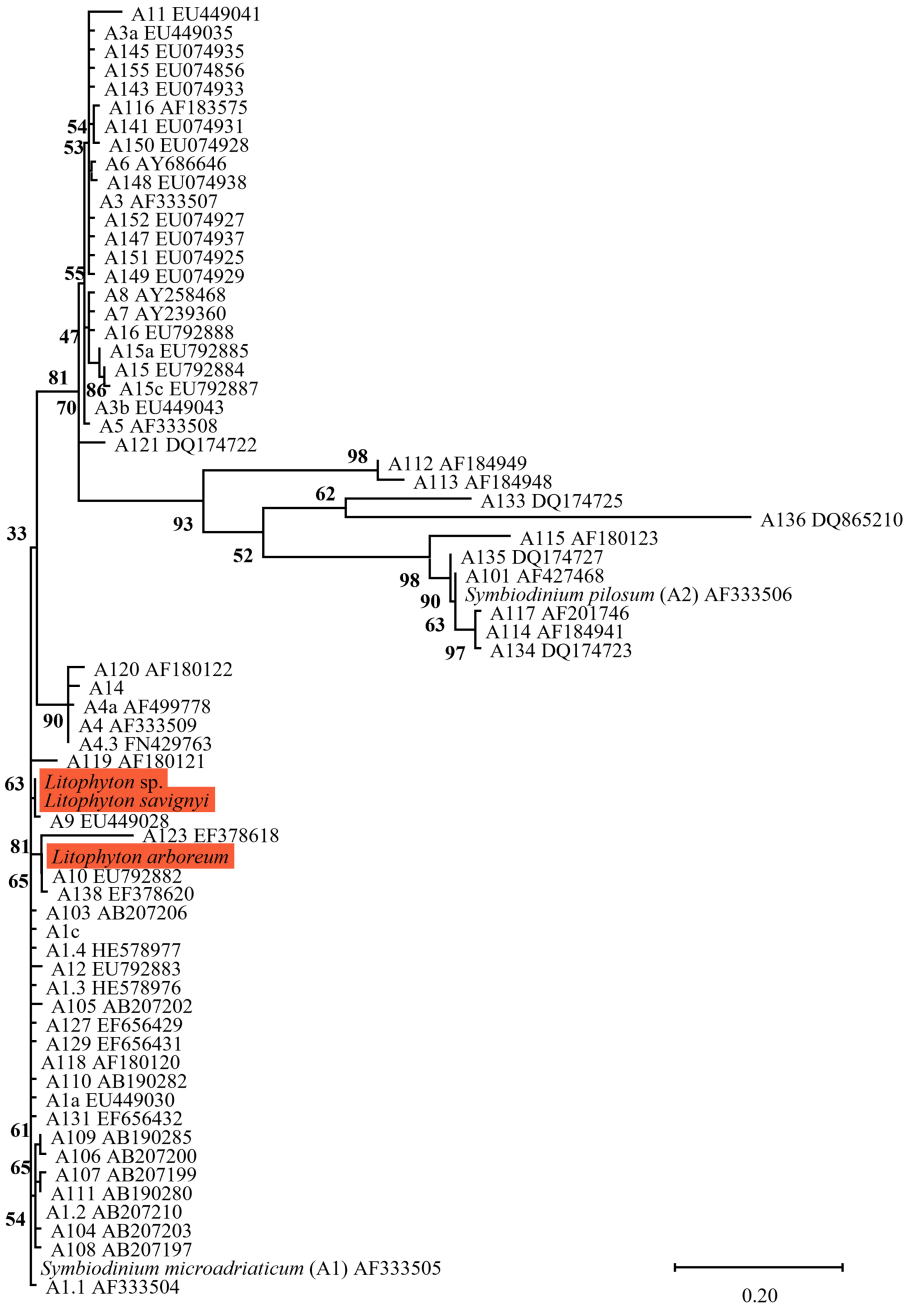
Phylogenetic relationship of the genus *Symbiodinium* (previously named clade A) based on ITS2 rDNA sequences (270 bp), under the K2P + G4 model. The maximum likelihood tree was reconstructed with IQ-TREE 2.1.3. ML bootstrap supports based on 1,000 replicates are indicated either above or below the corresponding branch. Orange color indicates new Symbiodiniaceae sequences that were obtained in the current study, for which the host name is indicated rather than the algal genetic type.

**Figure 3:**
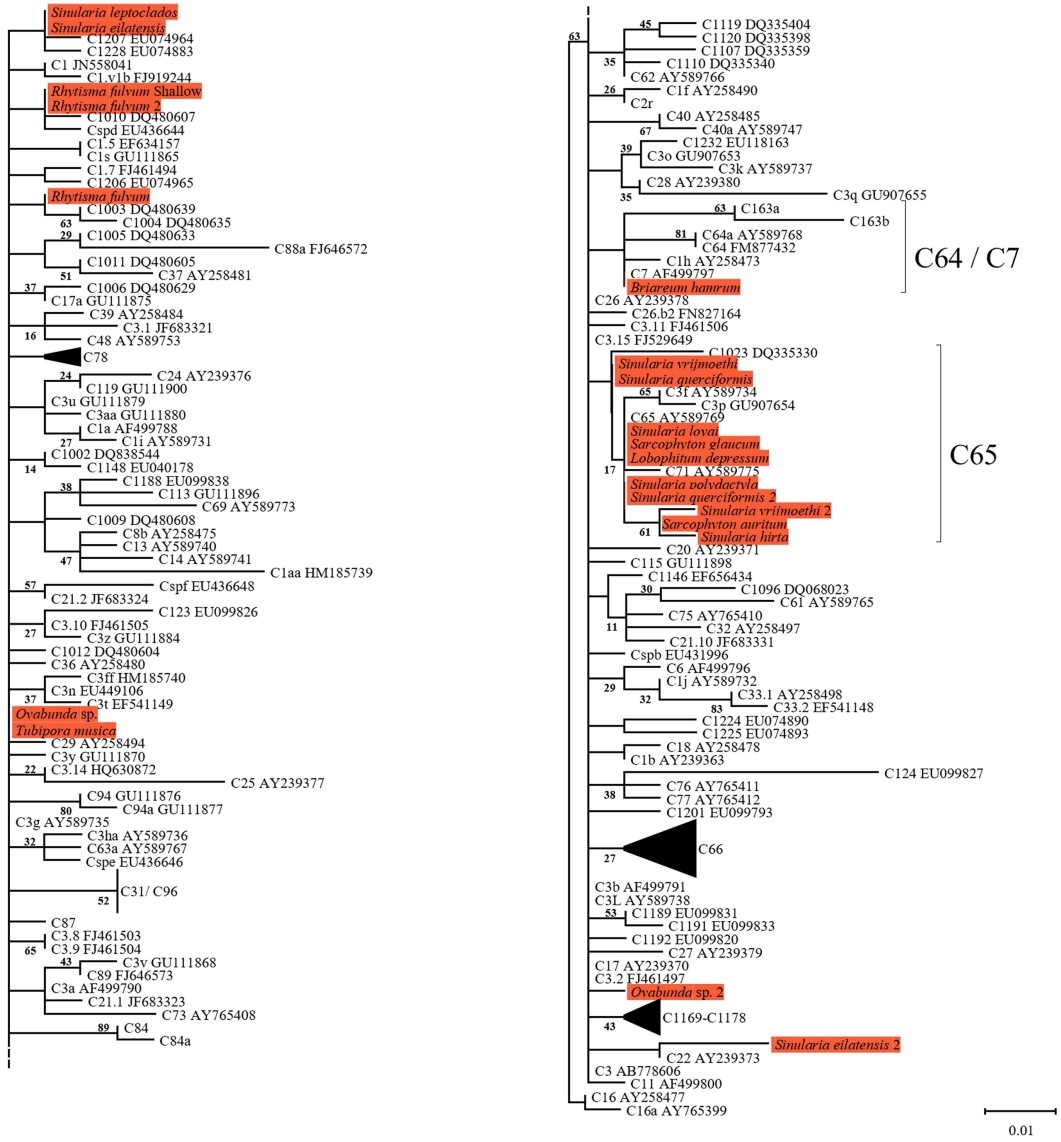
Right and Left; Phylogenetic relationship of the genus *Cladocopium* (previously named clade C) based on ITS2 rDNA sequences (270 bp), under the HKY + F model. The maximum likelihood tree was reconstructed with IQ-TREE 2.1.3. ML bootstrap supports based on 1,000 replicates are indicated either above or below the corresponding branch. Orange color indicates new Symbiodiniaceae sequences that were obtained in the current study for which the host name is indicated rather than the algal genetic type. Species that presented a variation in the obtained sequence are indicated by the number 2.

**Figure 4:**
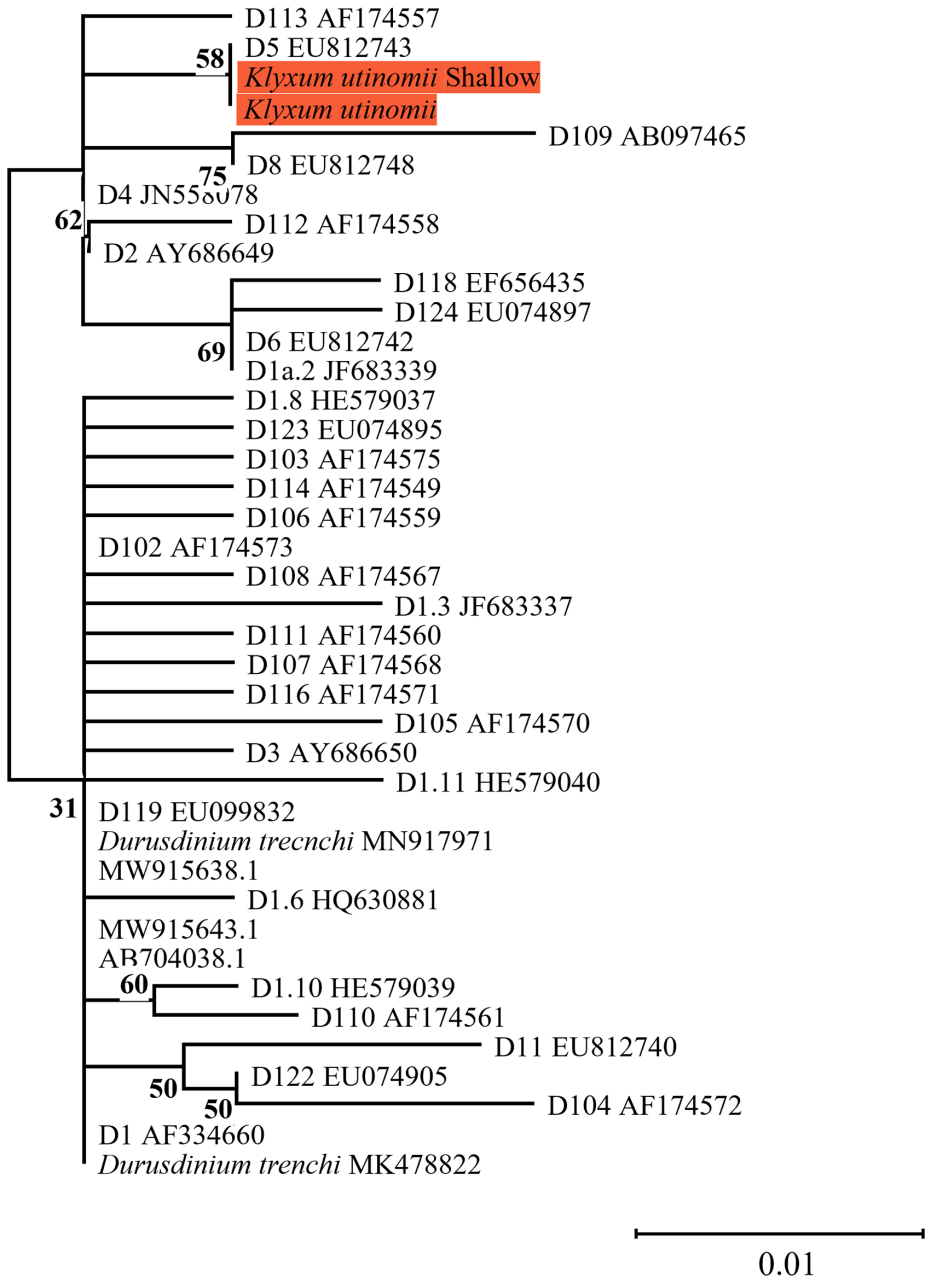
Phylogenetic relationship of the genus *Durusdinium* (previously named clade D) based on ITS2 rDNA sequences (270 bp), under the K2P model. The maximum likelihood tree was reconstructed with IQ-TREE 2.1.3. ML bootstrap supports based on 1,000 replicates are indicated either above or below the corresponding branch. Orange color indicates new Symbiodiniaceae sequences that were obtained in the current study for which the host name is indicated rather than the algal genetic type.

The same major Symbiodiniaceae lineages were identified based on both ITS2 and *cp23S* sequences. Most MCE octocorals were found to host Symbiodiniaceae of the genus *Cladocopium* (16/20), while *Litophyton arboreum*, *L*. *savignyi* and *Litophyton* sp. were associated with members of the genus *Symbiodinium*, and only one species, *Klyxum utinomii*, was associated with members of the genus *Durusdinium* (Figures 1–4). As expected, there was a marked difference in the phylogenetic resolution of the two gene regions, mainly visible in the analyses of the *Cladocopium* lineages (Figures 1, 3). The 19 *cp23S Cladocopium* sequences were all identical to the *Cladocopium* C90 sequence (JN557975), except for five sequences found in *Cladiella* sp., *R*. *fulvum*, *Ovabunda* sp., *Tubipora musica*, and *Xenia* sp., which presented several additional substitutions (Figure 1). In contrast, analyses of the 31 ITS2 sequences revealed the presence of 12 *Cladocopium* types in 14 different MCE octocoral species (Figure 3). Five species harbored more than one ITS2 type: *Ovabunda* sp., *R*. *fulvum*, S. *eilatensis S*. *querciformis*, and *S*. *vrijmoethi*. Interestingly, different members of *Cladocopium* were found between the two *Ovabunda* sp. samples, similar to the results obtained when using the *cp23S* marker (Figure 3: indicated by numbers). Only a few ITS2 sequences were found to contain a previously sequenced ITS2 type: *Ovabunda* sp. and *Tubipora musica*, which shared the same type and were found to be identical to the C3 type (AB778606). Similarly, *Lobophytum depressum*, *S*. *glaucum*, *S*. *loyai*, *S*. *polydactyla*, and *S*. *querciformis* presented a sequence identical to type C65 (AY589775). Finally, *Briareum hamrum* contained a *Cladocopium* type identical to C7 (AF499797). All other sequences were found to be new types (Figure 3).

### Symbiodiniaceae community structure

**Figure 5:**
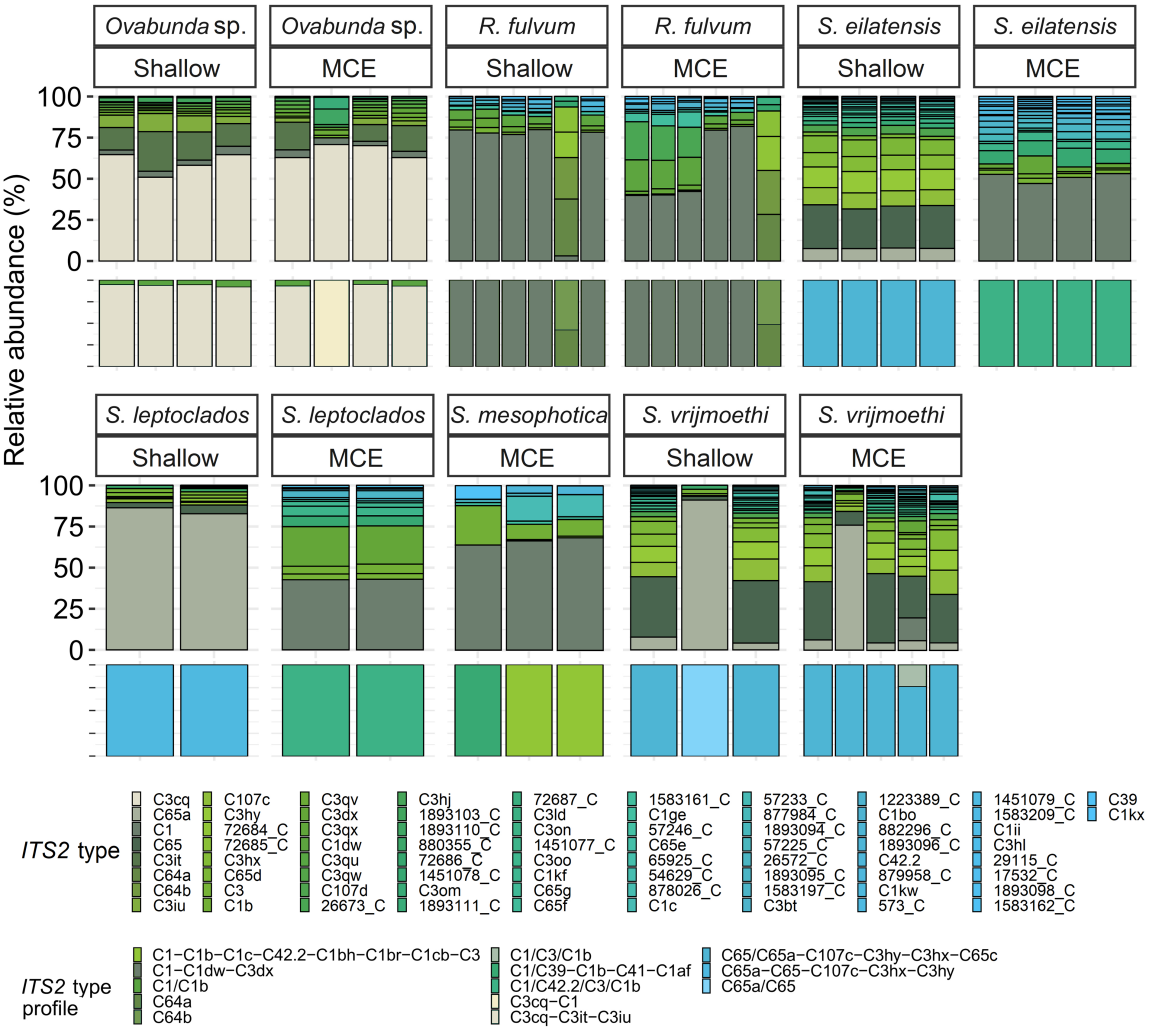
Normalized relative abundance of members of *Cladocopium* in octocoral host species from shallow and mesophotic reefs in the Gulf of Aqaba/Eilat. Relative abundances of ITS2 sequences and predicted ITS2 type-profiles (above and below, respectively) arranged by octocoral taxon and depth zone. Only the >0.01% most abundant sequences are displayed (*n* = 74). Sequences not used in the definition of ITS2 type profiles are given a unique database ID number followed by a letter referring to the genus the sequence is from (e.g., 29,115_C is a sequence from the genus *Cladocopium* with the database ID 29115). Type-profile names are listed according to the sequence of their abundance, with ‘/’ separating type-sequences with co-dominance in a given sample. *R., Rhytisma; S., Sinularia*.

**Figure 6:**
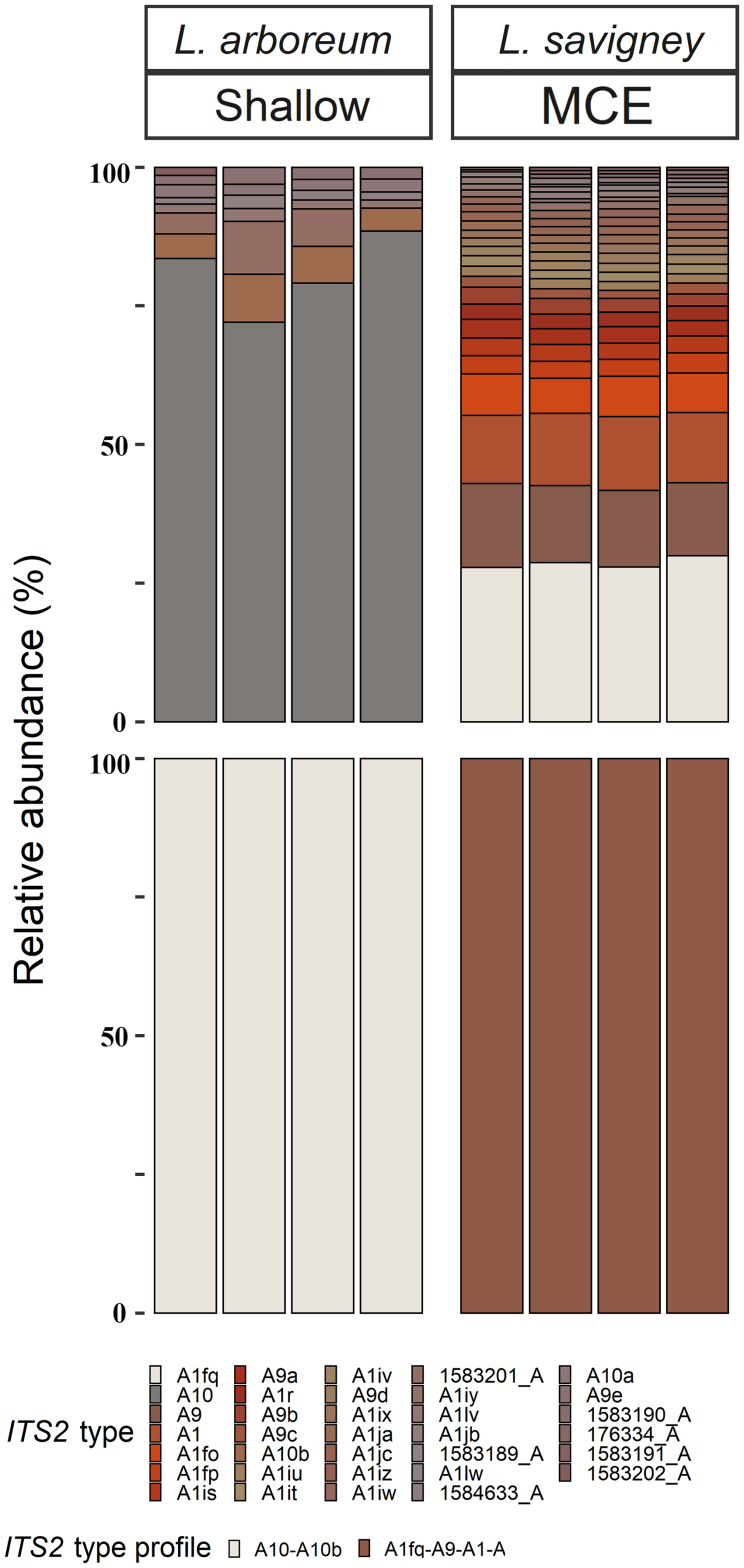
Normalized relative abundance of members of *Symbiodinium* in octocoral host species from shallow and mesophotic zones in the Gulf of Aqaba/Eilat. Relative abundances of ITS2 sequences and predicted ITS2 type-profiles (above and below, respectively) arranged by octocoral species and depth zone. Only the >0.01% most abundant sequences are displayed (*n* = 33). Sequences not used in the definition of ITS2 type profiles are given a unique database ID number followed by a letter referring to the genus the sequence is from (e.g., 1,583,189_A is a sequence from the genus *Symbiodinium* with the database ID 1583189).

All octocoral species were found to harbor symbiotic algae from a single genus (Figures 5, 6). The ITS2 type found in octocorals using high-throughput ITS2 sequencing were mostly comparable with the dominant ITS2 type found using Sanger sequencing, however some differences were also found (Figures 5, 6). Using both sequencing methods, the two examined *Litophyton* species harboring members of *Symbiodinium* were found to associate with distinct types (A10 in *L*. *arboreum*, A9 in *L*. *savignyi*). In the phylogenetic analyses, however, the dominant sequence obtained with Sanger sequencing was not always identical to the algal type identified using high-throughput ITS2. For example, one of the MCE *Ovabunda* sp. colonies was inferred to harbor C3 sequences using Sanger sequencing while C3cq was the dominant type with high-throughput ITS2 sequencing (Figures 2, 5). Similarly, the most abundant sequences in *R*. *fulvum*, *S*. *eilatensis* MCE, and *S*. *leptoclados* MCE colonies was identified as C1 using high-throughput sequencing, while the sequences obtained using Sanger contained a few ambiguous characters and thus differed from C1 (Figure 3). Moreover, in four out of five *S*. *vrijmoethi* MCE colonies, the most abundant type was C65, while in the Sanger sequencing, one of the sequence of *S*. *vrijmoethi* correspond to C65a. These differences stem from the fact that the sequence obtained using Sanger sequencing is a consensus of the different ITS2 types (weighted by the frequency of each type), and as such it can contain ambiguities and slightly differ from the major sequence.

SymPortal analysis of all high-throughput ITS2 sequences suggested that there are 15 distinct octocoral-associated type-profiles. Of these profiles, the majority were distinct *Cladocopium* ones (13 out of 15, recovered from 43 samples from six species). Most octocoral species were found to associate with a single type-profile (41/51). In contrast, seven *Ovabunda* sp. colonies, two *R*. *fulvum* colonies, and one *S*. *vrijmoethi* colony harbored an additional type-profile (Figure 5).

The nested-PERMANOVA test based on the ITS2 type sequences indicated that the Symbiodiniaceae community structure significantly varied among octocoral species (Table 2; *F* = 11.502, *p* = 0.001), but not across depth (Table 2; *F* = 1.466, *p* = 0.175). Despite the overall lack of effect of depth on the community structure, some nested-PERMANOVA analyses of the octocoral species *S*. *eilatensis* indicated significant differences also across depth (Table 2; *F* = 175.7, *p* = 0.024). More specifically, a pairwise PERMANOVA test of the community structure found in different host species indicated that *R*. *fulvum* and *Ovabunda* sp. consistently hosted different lineages to those of all *Cladocopium* host species; and *S*. *mesophotica* harbored different lineages to those of *S*. *vrijmoethi* and *S*. *eilatensis* (Table 3). Similarly, *L*. *arboreum* and *L*. *savignyi* hosted significantly different *Symbiodinium* communities from each other (Table 3). An additional nested and pairwise PERMANOVA test using the ITS2 type profiles also revealed comparable results, with the only exception of *S*. *mesophotica* algal symbionts being also significantly different from *R*. *fulvum*, *S*. *leptoclados*, and *Ovabunda* sp. (Supplementary Table S2).

**Table 2:** Results of permutational multivariate analysis of variance of octocoral species harboring *Cladocopium* ITS2 types in both shallow and MCE environments.

Statistical test	Compared taxa	Compared variable	*n*	Sum of squares	F model	R2	df	*p* value
Nested-PERMANOVA	All species	Species	40	7.863	11.502	0.568	4	0.001
Nested-PERMANOVA	All species	Depth	40	0.051	1.466	0.030	1	0.175
PERMANOVA	*Ovabunda* sp.	Depth	8	0.041	2.491	0.293	1	0.102
PERMANOVA	*R*. *fulvum*	Depth	12	0.129	0.743	0.069	1	0.543
PERMANOVA	*S*. *leptoclados*	Depth	4	0.973	185.646	0.989	1	0.333
PERMANOVA	*S*. *vrijmoethi*	Depth	8	0.048	0.287	0.046	1	0.768
PERMANOVA	*S*. *eilatensis*	Depth	8	1.890	175.702	0.967	1	0.024

Tests were based on Bray–Curtis dissimilarity distances and 999 permutations. All species were tested using a nested approach using depth or species taxon as a blocking factor. R., *Rhytisma; S*., *Sinularia*.

**Table 3:** Results of pairwise permutational multivariate analysis of variance of octocoral species harboring either *Cladocopium* or *Symbiodinium* Symbiodiniaceae ITS2 types.

Compared taxa	Sum of squares	F model	R2	df	*p* value	*p* adjusted
*S*. *mesophotica* vs. *S*. *vrijmoethi*	1.741	14.428	0.6158468	1	0.010	0.019
*S*. *mesophotica* vs. *S*. *leptoclados*	0.544	2.642	0.3457541	1	0.191	0.191
*S*. *mesophotica* vs. *R*. *fulvum*	0.329	2.243	0.1471529	1	0.041	0.054
*S*. *mesophotica* vs. *S*. *eilatensis*	0.768	3.458	0.2775578	1	0.024	0.038
*S*. *mesophotica* vs. *Ovabunda* sp.	1.740	85.070	0.9043263	1	0.004	0.009
*S*. *vrijmoethi* vs. *S*. *leptoclados*	0.819	4.043	0.2878875	1	0.043	0.054
*S*. *vrijmoethi* vs. *R*. *fulvum*	3.182	19.720	0.5228012	1	0.001	0.004
*S*. *vrijmoethi* vs. *S*. *eilatensis*	0.703	3.286	0.1900730	1	0.058	0.067
*S*. *vrijmoethi* vs. *Ovabunda* sp.	3.351	39.735	0.7394638	1	0.001	0.004
*S*. *leptoclados* vs. *R*. *fulvum*	0.826	4.063	0.2249273	1	0.025	0.038
*S*. *leptoclados* vs. *S*. *eilatensis*	0.473	1.609	0.1385694	1	0.142	0.152
*S*. *leptoclados* vs. *Ovabunda* sp.	1.765	15.711	0.6110578	1	0.003	0.007
*R*. *fulvum* vs. *S*. *eilatensis*	1.225	5.777	0.2429600	1	0.002	0.006
*R*. *fulvum* vs. *Ovabunda* sp.	3.433	30.863	0.6316250	1	0.001	0.004
*S*. *eilatensis* vs. *Ovabunda* sp.	2.686	17.955	0.5618906	1	0.001	0.004
*L*. *arboreum* vs. *L.* *savignyi*	1.89	87.46	0.93	1	0.0275	0.0275

Tests were based on Bray–Curtis dissimilarity distances and 999 permutations. R., *Rhytisma*; *S*., *Sinularia*.

SIMPER analysis comparing among the host species harboring members of *Cladocopium* revealed that several ITS2 types were responsible for most of the dissimilarity among host species. For example, C3cq and C3it accounted for 17–22% and 7–10% of the dissimilarity between *Ovabunda* sp. and the other host species, respectively. Similarly, C65 and C65a, were found in high abundance in *S*. *vrijmoethi* and *S*. *leptoclados* and accounted for 5–9% and 11–15% of the dissimilarity between these and the other species, respectively (Supplementary Table S1). SIMPER analysis between the two *Litophyton* species revealed that A10, which was the most abundant algal sequence in the shallow *L*. *arboreum*, accounted for 16% of the dissimilarity between both species’ ITS2 sequences. Conversely, A1, A1fq, and A9, which were found in *L*. *savignyi*, accounted for 5, 8, and 6% of the dissimilarity in the Symbiodiniaceae communities between both species’ ITS2 types, respectively (Supplementary Table S2). SIMPER analysis between ITS2 types in shallow and MCE *S*. *eilatensis* colonies indicated that C1, which was found only in the MCE colonies, accounted for almost 8% of the total difference; while both C65 and C65a, which were only found in the shallow colonies, accounted for 8 and 4% of the dissimilarity, respectively (Supplementary Table S3).

## Discussion

The current study has revealed that *Cladocopium* (previously known as clade C) is the most abundant Symbiodiniaceae in the MCE octocorals in the Gulf of Aqaba/ Eilat (Barneah et al., 2004; Goulet et al., 2008), which is in agreement with previous studies on the algal symbionts of shallow octocorals there. Examination of these symbionts in marine organisms across the Red Sea has highlighted the distinctive diversity found in the Gulf of Aqaba/Eilat and demonstrated that members of *Cladocopium* are the dominant algal symbionts there. This is in contrast to the central and southern Red Sea, where members of the genus *Symbiodinium* are the most common algal symbionts (i.e., stony corals: Sawall et al., 2014, Terraneo et al., 2019; zooanthid: Reimer et al., 2017; giant clam: Rossbach et al., 2021). The dominance of *Cladocopium* over *Symbiodinium* in the Gulf of Aqaba/Eilat has been suggested to be driven by the prevailing cooler water temperature and high salinity there, which differ from those in the other parts of the Red Sea (Sawall et al., 2014).

Three main factors have been suggested to affect the Symbiodiniaceae-type present in a coral holobiont: the cnidarian host species, light intensity (i.e., water depth) and seawater temperature (Cooper et al., 2011; Tonk et al., 2013; Reimer et al., 2017). Our results indicate that the host’s taxon plays a significant role in determining the octocoral-associated Symbiodiniaceae, while depth was found to significantly affect only the depth-generalist *S*. *eilatensis*. The degree of algal-specificity among cnidarian holobionts has also been suggested to be associated with the mode of symbiont acquisition, with strong specificity associated with vertical rather than horizontal transmission (Swain et al., 2018). Among octocorals, the relationships between algal-specificity and the mode of symbiont transmission is poorly understood, since to date it has only been recorded at the symbiont genus level (Barneah et al., 2004; Van Oppen et al., 2005; Goulet et al., 2019). In this work, we mainly investigated horizontal transmitters. The only vertical transmitter representatives are the members of the genus *Litophyton* (Benayahu et al., 1992; Goulet et al., 2008). Our results have revealed that distinct major *Symbiodinium* types are exclusively associated with each of the two *Litophyton* congeners (Figures 1, 2, 6; Supplementary Table S2). Out of the 37 *Symbiodinium* types identified in the two *Litophyton* congeners, only two minor ones were shared by both (i.e., A1iv and A1iW). In contrast, in the horizontal transmitters several *Cladocopium* types present in one host species are often found also in another species. As a case in point the dominant type C1 is not only present in congeners (e.g., S. *leptoclados S*. *vrijmoethi*., *S*. *eilatensis* MCE, and *S*. *mesophotica*) but also in species from different genera (i.e., *Ovabunda*, *Sinularia*, and *Rhytisma*). The same is true for C3, which is also present in high frequency in the members of both *Sinularia* and *Rhytisma* (Figure 5; Supplementary Table S1). Our results, obtained using high-throughput sequencing of the ITS2, thus provide for the first time evidence of a strong algal specificity in octocoral vertical transmitters, not only at the algal genus level but also at the ITS2 type profile resolution. C1 and C3 are ancestral sequence variants for major *Cladocopium* radiations which likely diversified more rapidly than the rate at which ITS2 accumulates mutations in this genus (Thornhill et al., 2014). Thus, even though the ITS2 sequence is shared across a variety of host species, a higher resolution marker such as *psbA* might indicate that in fact, different C1 lineages associate with different octocoral hosts (LaJeunesse and Thornhill, 2011). Furthermore, future research should attempt to understand the influence of members of the genus *Symbiodinium* on the dispersal, distribution, and photo-physiology of both *Litophyton* host species across a broad depth gradient.

Despite being dominant on many Red Sea and Indo-Pacific reefs, octocoral-symbiont associations have been poorly studied. Nonetheless, several comparisons between the current study and previous ones can be made. First, as suggested by Goulet et al. (2008), the current ITS2-type profile analysis indicated that octocorals mostly host a single population of symbionts. However, some exceptions were found, such as in most of the *Ovabunda* sp. colonies and in one colony of the MCE *S*. *vrijmoethi*, both of which were found to host an additional background population (Figure 5). Second, Osman et al. (2020) showed that ITS2 C65 variants associate with *Sarcophyton trocheliophorum* in the Gulf of Aqaba/Eilat as well as in other reefs of the northern Red Sea. This corresponds to our finding that eight species, including two *Sarcophyton* ones, associate with ITS2 type C65 or variants that are closely related, such as ITS2 C71 (Figures 3, 5). Interestingly, ITS2 C65 types have been previously reported to associate only with octocoral species (LaJeunesse et al., 2004; Goulet et al., 2008). In addition, ITS2 C71 has also only been found in *Sarcophyton* sp. from the South China Sea (Sanqiang et al., 2018). Furthermore, the dominant *Symbiodinium* ITS2 types found in the two *Litophyton* species in this study were found to be closely related to ITS2 sequences identified in other *Litophyton* colonies in the southern Red Sea (A9 EU449028 and A10 EU792882; Figure 2). These observations suggest that the association between octocorals and Symbiodiniaceae is not only at the genus level (see Goulet et al., 2008) but also at the lineage-level within a Symbiodiniaceae genus. The finding of specific octocoral-Symbiodiniaceae associations may be attributed to the different ‘micro-environment’ conditions in this group of organisms compared to those of scleractinians, such as reduced light amplification and dispersion by the skeleton (Kramer et al., 2022), a thick coenenchyma that limits gas and nutrient exchanges through the epidermal tissue, and the ability of octocorals to contract their tissue, thus preventing the symbionts from obtaining optimal exposure to light and nutrients (Fabricius and Klumpp, 1995; Ferrier-Pagès et al., 2021).

The depth-related zonation patterns of Symbiodiniaceae on coral reefs have been discussed in several studies examining stony-coral symbiont associations, highlighting certain aspects of the holobiont ecology and its response to changing environmental conditions (Frade et al., 2008a; Bongaerts et al., 2011, 2015; Cooper et al., 2011; Byler et al., 2013; Ziegler et al., 2015; Eckert et al., 2020). Our study is the first to examine the host-symbiont associations in ‘depth-generalist’ octocorals across their entire depth of occurrence. Specifically, our findings indicate that the shift in symbiont community composition across depth is species related and cannot be considered as a general phenomenon. The ‘depth-generalist’ *S*. *eilatensis* was found to harbor different ITS2-types at shallower depths compared to in the MCEs, whereas *S*. *vrijmoethi* displayed similar ITS2 sequences across depth (*S*. *eilatensis*:12–17 m: C65, C65a, C107, and C3hy; 60–67 m: C1, C1b, C39, and C41; *S*. *vrijmoethi:* C65, C65a, C107, and C3hy, Figure 5). Previous studies have suggested that while a shift in the symbiont community across depth may functionally benefit certain coral holobionts, this may incur metabolic costs (Frade et al., 2008a,b; Cooper et al., 2011). However, a recent study examining the physiological properties of three depth-generalist octocorals on Eilat’s reefs (i.e., *S*. *eilatensis*, *S*. *vrijmoethi*, and *S*. *leptoclados*), showed that all three exhibit a similar trend of lower photo-physiological capabilities and nitrogen assimilation with increased depth (Ferrier-Pagès et al., 2021). Therefore, it seems that, as in the case of *S*. *eilatensis*, the shift in symbiont community across depth might not impact the functional physiology of the colonies with increased depth, relative to the congener *S*. *vrijmoethi*. It is possible, therefore, that the change in symbiont community structure of *S*. *eilatensis* has little or no functional significance and is, alternatively, driven by the host’s distribution and factors such as species lineage and reproduction (Frade et al., 2008a). Nonetheless, in light of the small sample size, caution must be applied, as the findings might not relate evenly to all the studied species. For example, although *S*. *leptoclados* colonies were found to host different ITS2-profile across depth (Figure 5), due to the small sample size (*n* = 2 for each depth habitat) this result was not found to be significant and awaits confirmation in future studies. In addition, three of the host species reported here were only found once during our collection surveys (Table 1), and therefore, although the information on their algal symbionts adds to the total diversity, further confirmation regarding their endosymbionts requires a larger sample size.

The current finding of *Durusdinium* type D5 in *K*. *utinomii* from both shallow and mesophotic colonies (Figure 4) is noteworthy. Symbionts of the genus *Durusdinium* (previously known as clade D) have been recorded in the warmer waters of the central and southern Red Sea (Sawall et al., 2014; Terraneo et al., 2019). It is commonly considered as ‘thermally tolerant’ (e.g., *Durusdinium trenchii*; Kennedy et al., 2015; Silverstein et al., 2017; Wham et al., 2017; but see Smith et al., 2017). However, several other *Durusdinium* types were found in scleractinian coral species from marginal habitats over a large latitudinal gradient while demonstrating high algal-specificity. The presence of *Durusdinium* in these species was suggested to be related to the vertical transmission of photosymbionts in these host corals (Lien et al., 2013; Lajeunesse et al., 2014). Future studies should examine the unique association between the *Klyxum* host and its *Durusdinium* symbionts, along with its possible selective advantages for the holobiont association complex.

In summary, despite being abundant in many reef ecosystems, Symbiodiniaceae associations with octocorals are still poorly understood, especially in regard to their diversity at the type-level in MCE habitats. The current study has revealed novel ITS2-types and profiles, previously not recorded in any other cnidarian-hosts. Additionally, we demonstrate that the diversity of these associations across depth, at the symbiont-lineage level, is likely to be driven by a strong algal-specificity. Such distinctive associations probably represent a co-diversification between the octocoral hosts and their micro-algae and, therefore, a thorough examination of these associations, using an NGS approach, may well yield further insights into the evolution and ecology of octocoral symbiosis with their photosymbionts.

## Data availability statement

The Sanger sequences of the ITS2 and *cp23s* markers are deposited in the GenBank database (National Center for Biotechnology Information, NCBI), under accession numbers OP120823–OP120848 and OP135806-OP135828. The Illumina sequences of the ITS2 marker are deposited under the biosample accessions SAMN30076298-SAMN30076348 at NCBI. The raw data, the sequence alignments, and the R scripts used in this work can be found at https://figshare.com/articles/dataset/CE-Octocoral-microalgae/20407596/1.

## Author contributions

RL led the study, collected the samples, performed the laboratory work, analyzed the data, and wrote the first draft. DH supported and facilitated the laboratory work, and supervised the data analysis. YB and DH co-supervised the study, provided the resources, and edited the manuscript. All authors contributed to the article and approved the submitted version.

## Funding

This research was supported by Horizon 2020 EU grant GA n° 634674 Project Acronym: TASCMAR- Tools and strategies to access to original bioactive compounds from cultivated marine invertebrates and associated symbionts. DH acknowledges the support of Israel Science Foundation (Grant Number 652/20).

## Conflict of interest

The authors declare that the research was conducted in the absence of any commercial or financial relationships that could be construed as a potential conflict of interest.

## Publisher’s note

All claims expressed in this article are solely those of the authors and do not necessarily represent those of their affiliated organizations, or those of the publisher, the editors and the reviewers. Any product that may be evaluated in this article, or claim that may be made by its manufacturer, is not guaranteed or endorsed by the publisher.

## Footnotes

^1^http://guidance.tau.ac.il/, last accessed on January 21, 2021

^2^https://github.com/RyanEckert/Belize_Mcav_Symbiodiniaceae_ITS2, last accessed on December 20, 2021
