# Supplementary material for: Octocorals in the Gulf of Aqaba exhibit high photosymbiont fidelity
Source: Front Microbiol. 2022 Nov 25;13:1005471. doi: 10.3389/fmicb.2022.1005471 (PMC9732034; doi:10.3389/fmicb.2022.1005471)
Supplement: Supplementary file 1 [file Data_Sheet_1.docx]

Supplementary

Octocorals in the Gulf of Aqaba exhibit high symbiont fidelity

Ronen Liberman^1,2^, Dorothée Huchon^1,3^, and Yehuda Benayahu^1^

^1^ School of Zoology, The George S. Wise Faculty of Life Sciences, Tel-Aviv University, Tel-Aviv 69978, Israel.

^2^ The Interuniversity Institute for Marine Sciences, Eilat, 8810302, Israel.

^3^ The Steinhardt Museum of Natural History and National Research Center, Tel Aviv University, Tel Aviv, Israel

**Corresponding Author**
Ronenliberman@gmail.com

Supplementary Table 1. Similarity percentage (SIMPER) test results of the significant *Cladocopium* ITS2-types between species pairs. R.=*Rhytisma* and S.= *Sinularia*

| Species compared | ITS2 type | Average contribution | Average abundance species 1 | Average abundance species 2 | *p*. value |
| --- | --- | --- | --- | --- | --- |
| *S. vrijmoethi vs. S. leptoclados* | C65a | 0.13 | 591.08 | 881.13 | 0.04 |
| *S. vrijmoethi vs. Ovabunda* sp*.* | C3iu | 0.04 | 0.00 | 388.67 | < 0.01 |
| *S. vrijmoethi vs. S. leptoclados* | C65 | 0.06 | 571.23 | 194.63 | 0.03 |
| *S. vrijmoethi vs. S. leptoclados* | C3hy | 0.03 | 282.71 | 72.74 | 0.04 |
| *S. vrijmoethi vs. S. leptoclados* | C107d | 0.02 | 160.65 | 0.00 | 0.01 |
| *S. vrijmoethi vs. R. fulvum* | C65a | 0.11 | 591.08 | 0.00 | < 0.01 |
| *S. vrijmoethi vs. R. fulvum* | C65 | 0.10 | 571.23 | 0.00 | < 0.01 |
| *S. vrijmoethi vs. R. fulvum* | C1 | 0.07 | 54.68 | 455.54 | < 0.01 |
| *S. vrijmoethi vs. R. fulvum* | C3hx | 0.05 | 270.60 | 0.00 | < 0.01 |
| *S. vrijmoethi vs. R. fulvum* | C107c | 0.05 | 295.08 | 0.00 | < 0.01 |
| *S. vrijmoethi vs. R. fulvum* | C3hy | 0.05 | 282.71 | 0.00 | < 0.01 |
| *S. vrijmoethi vs. R. fulvum* | C65d | 0.04 | 248.64 | 0.00 | < 0.01 |
| *S. vrijmoethi vs. R. fulvum* | C107d | 0.03 | 160.65 | 0.00 | < 0.01 |
| *S. vrijmoethi vs. R. fulvum* | C1dw | 0.03 | 0.00 | 160.75 | 0.01 |
| *S. vrijmoethi vs. R. fulvum* | C3 | 0.02 | 201.24 | 56.86 | < 0.01 |
| *S. vrijmoethi vs. Ovabunda* sp*.* | C3cq | 0.17 | 0.00 | 1,481.45 | < 0.01 |
| *S. vrijmoethi vs. Ovabunda* sp*.* | C3it | 0.07 | 0.00 | 646.47 | < 0.01 |
| *S. vrijmoethi vs. Ovabunda* sp*.* | C65 | 0.06 | 571.23 | 0.00 | < 0.01 |
| *S. vrijmoethi vs. Ovabunda* sp*.* | C1b | 0.03 | 16.14 | 241.97 | < 0.01 |
| *S. vrijmoethi vs. Ovabunda* sp*.* | C65d | 0.03 | 248.64 | 0.00 | 0.01 |
| *S. vrijmoethi vs. Ovabunda* sp*.* | C3hx | 0.03 | 270.60 | 0.00 | 0.01 |
| *S. vrijmoethi vs. Ovabunda* sp*.* | C107c | 0.03 | 295.08 | 0.00 | 0.01 |
| *S. vrijmoethi vs. Ovabunda* sp*.* | C3hy | 0.03 | 282.71 | 0.00 | 0.01 |
| *S. vrijmoethi vs. Ovabunda* sp*.* | C3 | 0.02 | 201.24 | 0.00 | < 0.01 |
| *S. vrijmoethi vs .S. eilatensis* | C65 | 0.05 | 571.23 | 246.12 | 0.03 |
| *S. leptoclados vs. S. eilatensis* | C65a | 0.13 | 881.13 | 134.71 | 0.04 |
| *S. leptoclados vs. R. fulvum* | C65a | 0.16 | 881.13 | 0.00 | < 0.01 |
| *S. leptoclados vs. R. fulvum* | C1 | 0.05 | 215.78 | 455.54 | 0.02 |
| *S. leptoclados vs. R. fulvum* | C1dw | 0.04 | 0.00 | 160.75 | < 0.01 |
| *S. leptoclados vs. R. fulvum* | C3dx | 0.04 | 160.40 | 92.33 | < 0.01 |
| *S. leptoclados vs. R. fulvum* | C3 | 0.03 | 201.96 | 56.86 | < 0.01 |
| *S. leptoclados vs. R. fulvum* | 1451078_C | 0.02 | 82.58 | 0.00 | 0.01 |
| *S. leptoclados vs. Ovabunda* sp*.* | C3cq | 0.20 | 0.00 | 1,481.45 | < 0.01 |
| *S. leptoclados vs. Ovabunda* sp*.* | C3it | 0.09 | 0.00 | 646.47 | < 0.01 |
| *S. leptoclados vs. Ovabunda* sp*.* | C3 | 0.03 | 201.96 | 0.00 | < 0.01 |
| *S. leptoclados vs. Ovabunda* sp*.* | C1b | 0.02 | 75.33 | 241.97 | 0.03 |
| *S. leptoclados vs .S. eilatensis* | C3dx | 0.03 | 160.40 | 76.45 | 0.01 |
| *S. eilatensis vs. Ovabunda* sp*.* | C3cq | 0.19 | 0.00 | 1,481.45 | < 0.01 |
| *S. eilatensis vs. Ovabunda* sp*.* | C3it | 0.08 | 0.00 | 646.47 | < 0.01 |
| *S. eilatensis vs. Ovabunda* sp*.* | C3iu | 0.05 | 0.00 | 388.67 | < 0.01 |
| *S. eilatensis vs. Ovabunda* sp*.* | C1b | 0.03 | 43.72 | 241.97 | < 0.01 |
| *R. fulvum vs. S. eilatensis* | C1 | 0.05 | 455.54 | 251.52 | 0.02 |
| *R. fulvum vs. S. eilatensis* | 1451078_C | 0.03 | 0.00 | 108.07 | < 0.01 |
| *R. fulvum vs. S. eilatensis* | C65d | 0.03 | 0.00 | 154.39 | 0.02 |
| *R. fulvum vs. S. eilatensis* | C3hx | 0.03 | 0.00 | 144.75 | 0.04 |
| *R. fulvum vs. S. eilatensis* | C1dw | 0.03 | 160.75 | 0.00 | < 0.01 |
| *R. fulvum vs. S. eilatensis* | C3hy | 0.03 | 0.00 | 173.33 | 0.01 |
| *R. fulvum vs. Ovabunda* sp*.* | C3cq | 0.23 | 0.00 | 1,481.45 | < 0.01 |
| *R. fulvum vs. Ovabunda* sp*.* | C3it | 0.10 | 0.00 | 646.47 | < 0.01 |
| *R. fulvum vs. Ovabunda* sp*.* | C1b | 0.04 | 0.00 | 241.97 | < 0.01 |
| *R. fulvum vs. Ovabunda* sp*.* | C1dw | 0.03 | 160.75 | 0.00 | 0.03 |

Supplementary Table 2. Similarity percentage (SIMPER) test results of the significant *Symbiodinium* ITS2-types between *Litophyton* species pairs. L= *Litophyton*.

| Species compared | ITS2 type | Average contribution | Average abundance species 1 | Average abundance species 2 | *p*. value |
| --- | --- | --- | --- | --- | --- |
| *L. arboreum vs. L. savignyi* | A10 | 0.16 | 105.49 | 0.00 | 0.03 |
| *L. arboreum vs. L. savignyi* | A1fq | 0.08 | 0.00 | 53.49 | 0.03 |
| *L. arboreum vs. L. savignyi* | A9 | 0.06 | 0.00 | 37.29 | 0.03 |
| *L. arboreum vs. L. savignyi* | A1 | 0.05 | 0.00 | 35.76 | 0.03 |
| *L. arboreum vs. L. savignyi* | A10b | 0.04 | 28.73 | 0.00 | 0.03 |
| *L. arboreum vs. L. savignyi* | A1fo | 0.04 | 0.00 | 26.49 | 0.03 |
| *L. arboreum vs. L. savignyi* | A10a | 0.03 | 17.36 | 0.00 | 0.03 |
| *L. arboreum vs. L. savignyi* | A9e | 0.03 | 17.56 | 0.00 | 0.03 |
| *L. arboreum vs. L. savignyi* | A9a | 0.03 | 0.00 | 17.25 | 0.03 |
| *L. arboreum vs. L. savignyi* | A1is | 0.03 | 0.00 | 17.38 | 0.03 |
| *L. arboreum vs. L. savignyi* | A1fp | 0.03 | 0.00 | 18.06 | 0.03 |
| *L. arboreum vs. L. savignyi* | A9b | 0.02 | 0.00 | 16.07 | 0.03 |
| *L. arboreum vs. L. savignyi* | A1r | 0.02 | 0.00 | 16.28 | 0.03 |

Supplementary Table 3. Similarity percentage (SIMPER) test results for the Cladocopium ITS2-types significantly contributing to the differences found in Sinularia eilatensis between shallow and MCE colonies.

| Species compared | ITS2 type | Average contribution | Average abundance shallow | Average abundance MCE | *p*. value |
| --- | --- | --- | --- | --- | --- |
| Shallow *vs.* MCE | C65 | 0.08 | 492.25 | 0.00 | 0.04 |
| Shallow *vs.* MCE | C1 | 0.08 | 0.00 | 503.05 | 0.04 |
| Shallow *vs.* MCE | C3hy | 0.06 | 346.66 | 0.00 | 0.04 |
| Shallow *vs.* MCE | C65d | 0.05 | 308.78 | 0.00 | 0.04 |
| Shallow *vs.* MCE | C3hx | 0.05 | 289.51 | 0.00 | 0.04 |
| Shallow *vs.* MCE | C107c | 0.05 | 304.65 | 0.00 | 0.04 |
| Shallow *vs.* MCE | C65a | 0.04 | 269.43 | 0.00 | 0.04 |
| Shallow *vs.* MCE | C107d | 0.03 | 215.24 | 0.00 | 0.04 |
| Shallow *vs.* MCE | 1451078_C | 0.03 | 0.00 | 216.14 | 0.04 |
| Shallow *vs.* MCE | 1451077_C | 0.02 | 0.00 | 152.85 | 0.04 |
| Shallow *vs.* MCE | C3dx | 0.02 | 0.00 | 152.90 | 0.04 |

Supplementary table 4. Results of nested and pairwise permutational multivariate analysis of variance of octocoral species harboring *Cladocopium* ITS2-type profiles in both shallow and MCE environments. Tests were based on Bray-Curtis dissimilarity distances and 999 permutations. All species were tested using a nested approach using depth or species taxon as a blocking factor.

| Statistical test | Compared taxa | Compared variable | n | Sum of squares | F. Model | R2 | df | p. value |
| --- | --- | --- | --- | --- | --- | --- | --- | --- |
| Nested- PERMANOVA | All species | Species | 40 | 9.623 | 13.093 | 0.599 | 4 | 0.001 |
| Nested- PERMANOVA | All species | Depth | 40 | 0.5449 | 1.335 | 0.033 | 1 | 0.236 |
| Pairwise PERMANOVA | *S. mesophotica vs.  S. vrijmoethi* | ITS2 type profile | ̶ | 1.455 | 8.417 | 0.483 | 1 | 0.013 |
| Pairwise PERMANOVA | *S. mesophotica vs.  S. leptoclados* | ITS2 type profile | ̶ | 0.905 | 2.714 | 0.352 | 1 | 0.086 |
| Pairwise PERMANOVA | *S. mesophotica vs.  R. fulvum* | ITS2 type profile | ̶ | 1.533 | 8.541 | 0.397 | 1 | 0.002 |
| Pairwise PERMANOVA | *S. mesophotica vs.  S. eilatensis* | ITS2 type profile | ̶ | 1.152 | 3.886 | 0.302 | 1 | 0.054 |
| Pairwise PERMANOVA | *S. mesophotica vs. Ovabunda* sp*.* | ITS2 type profile | ̶ | 1.458 | 8.509 | 0.486 | 1 | 0.011 |
| Pairwise PERMANOVA | *S. vrijmoethi vs.  S. leptoclados* | ITS2 type profile | ̶ | 1.704 | 9.021 | 0.474 | 1 | 0.002 |
| Pairwise PERMANOVA | *S. vrijmoethi vs.  R. fulvum* | ITS2 type profile | ̶ | 3.600 | 25.355 | 0.585 | 1 | 0.000 |
| Pairwise PERMANOVA | *S. vrijmoethi vs.  S. eilatensis* | ITS2 type profile | ̶ | 0.810 | 3.927 | 0.219 | 1 | 0.079 |
| Pairwise PERMANOVA | *S. vrijmoethi vs. Ovabunda* sp*.* | ITS2 type profile | ̶ | 3.118 | 24.740 | 0.639 | 1 | 0.001 |
| Pairwise PERMANOVA | *S. leptoclados vs.  R. fulvum* | ITS2 type profile | ̶ | 1.833 | 9.623 | 0.407 | 1 | 0.001 |
| Pairwise PERMANOVA | *S. leptoclados vs.  S. eilatensis* | ITS2 type profile | ̶ | 0.667 | 2.222 | 0.182 | 1 | 0.140 |
| Pairwise PERMANOVA | *S. leptoclados vs. Ovabunda* sp*.* | ITS2 type profile | ̶ | 1.708 | 9.107 | 0.477 | 1 | 0.002 |
| Pairwise PERMANOVA | *R. fulvum vs.  S. eilatensis* | ITS2 type profile | ̶ | 2.933 | 14.398 | 0.444 | 1 | 0.000 |
| Pairwise PERMANOVA | *R. fulvum vs. Ovabunda* sp*.* | ITS2 type profile | ̶ | 3.608 | 25.541 | 0.587 | 1 | 0.000 |
| Pairwise PERMANOVA | *S. eilatensis vs. Ovabunda* sp*.* | ITS2 type profile | ̶ | 2.562 | 12.474 | 0.471 | 1 | 0.000 |


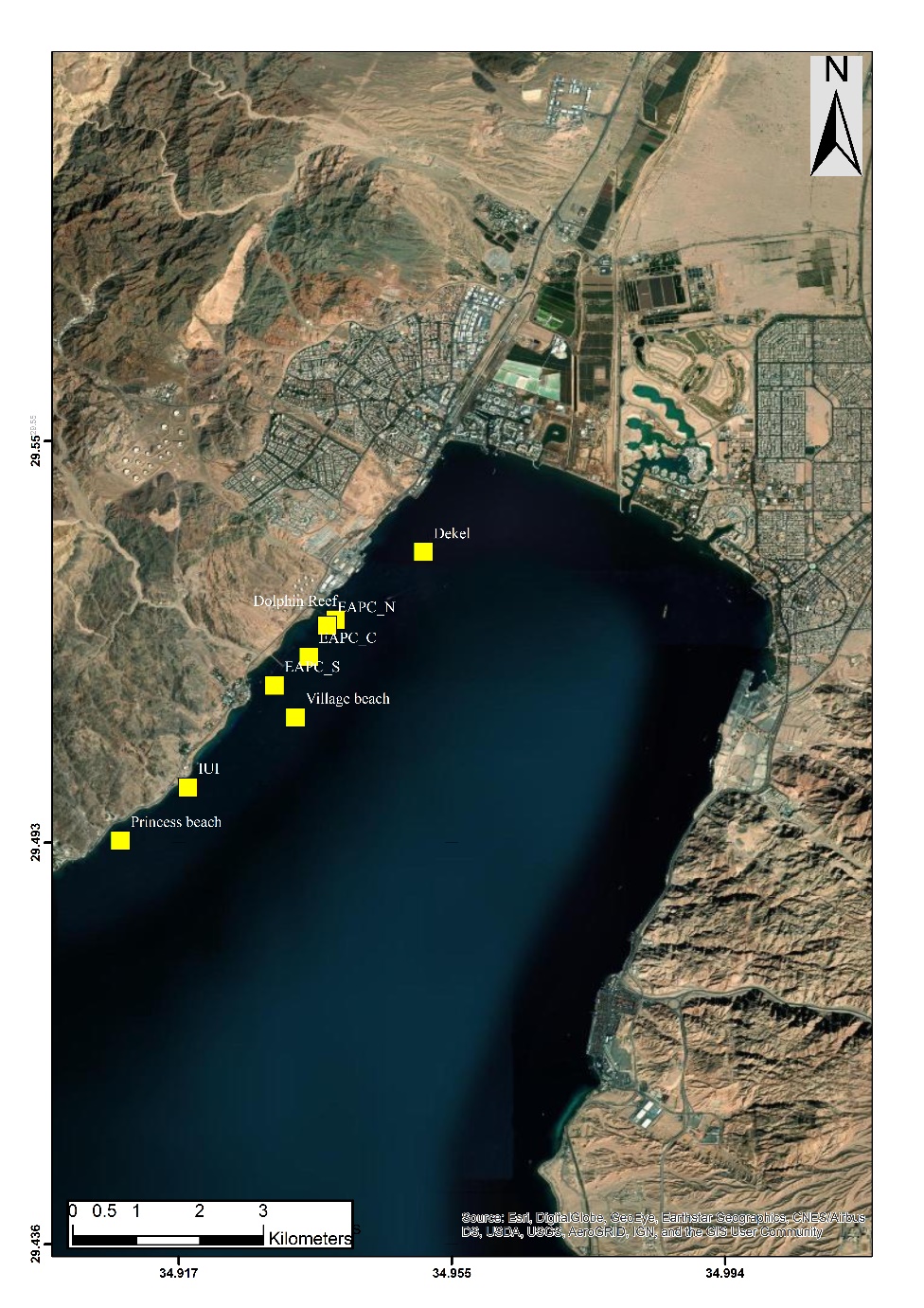


Figure S1. Sampling locations and specimen collection overview. Gulf of Aqaba/ Eilat Sea map with sampling localities.
