# Supplementary figures and images for: Octocorals in the Gulf of Aqaba exhibit high photosymbiont fidelity
Source: Front Microbiol. 2022 Nov 25;13:1005471. doi: 10.3389/fmicb.2022.1005471 (PMC9732034; doi:10.3389/fmicb.2022.1005471)

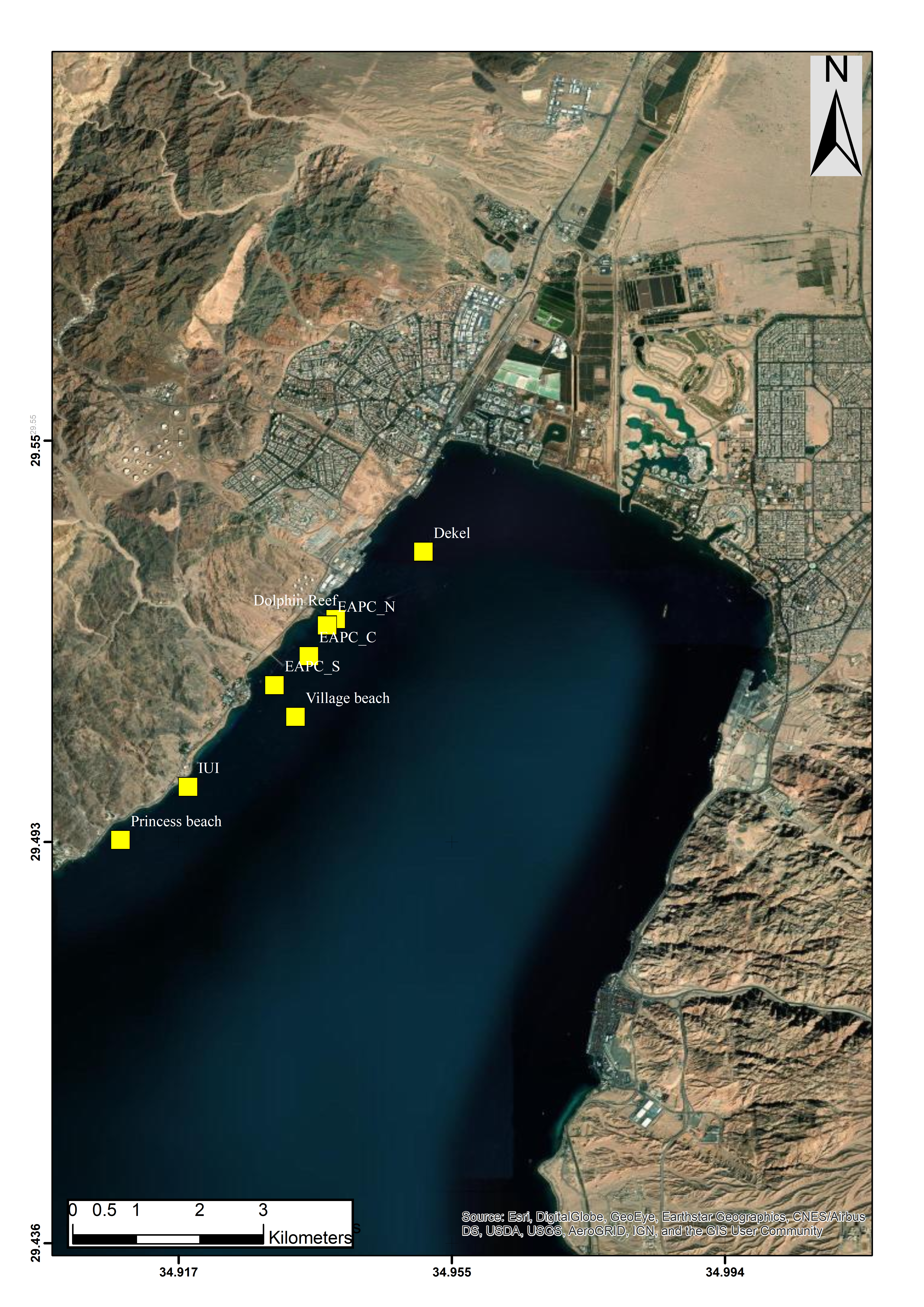

Supplement: Supplementary file 2 [file Image_1.PNG]
